# Acknowledgement to Reviewers of *Cancers* in 2013

**DOI:** 10.3390/cancers6010491

**Published:** 2014-02-28

**Authors:** Cancers Editorial Office

**Affiliations:** MDPI AG, Klybeckstrasse 64, CH-4057 Basel, Switzerland

The editors of *Cancers* would like to express their sincere gratitude to the following reviewers for assessing manuscripts in 2013:

Aft, Rebecca
Agaimy, Abbas
Al-Daccak, Reem
Allan, Allison
Antonarakis, Emmanuel
Aoki, Kazunori
Arafat, Hwyda A.
Awe, Julius A.
Bachelot, T.
Bagley, Rebecca G.
Bakhoum, Samuel F.
Baldassarre, Antonella
Balic, Marija
Barnett, Samuel L.
Becker, Minerva
Beckner, Marie E.
Bertolini, Francesco
Bianco-Miotto, Tina
Billings, Steven D.
Blanc, Jean-Frederic
Blandino, Giovanni
Bodelon, Clara
Bologna-Molina, R.
Brady-Kalnay, Susann M.
Breckpot, Karine
Brewer, Jerry D.
Brown, Tanya Renae
Brunner, Thomas
Buglione, Michela
Calvisi, Diego F.
Campbell, Daniel B.
Campbell, Moray
Canaani, Dan
Carey, Mariko
Carlberg, Carsten
Carmen, Garrido
Carra, Serena
Carrel, Thomas
Casanova, Llanos
Cassoni, Paola
Ceder, Yvonne
Chabner, Bruce A.
Chauchereau, Anne
Chen, Clark
Chen, Mu-Kuan
Chen, Thomas C.
Chen, Yi-Ting
Chevet, Eric
Choong, Peter F. M.
Chuang, Shu-Chun
Chuma, Makoto
Clark, Geoffrey J.
Clendenen, Tess V.
Conti, Alfredo
Cooper, Wendy N.
Corbí, Angel L.
Corso, Simona
Dumont, Jacques E.
Daniele, ssa Antonella
de Bruin, Alain
Decrock, Elke
DeLaney, Thomas F.
Domínguez, G.
Doyle, Gerald V.
Drouin, Régen
Dwyer-Nield, Lori D.
Eckardt, Jeffrey
Eilber, Fritz C.
Erkizan, Verda
Fan, Yihui
Fingleton, Barbara
Fiorica, F.
Fishman, Mayer
Fogliata, A.
Fortunati, N.
Fourkala, Evangelia-ourania
Fraczek, Joanna
Franchitto, A.
Frank, D.
Frohlich, Mark W.
Fuchs, Bruno
Galileo, Deni S.
Gebhardt, Rolf
Gehrmann, Mathias
Georgakilas, Alexandros G.
Gilles, Christine
Giordano, Silvia
Golemis, Erica A.
Gomez-Bougie, P.
Göndör, Anita
Gorges, Tobias M.
Görgün, Güllü
Gottschalk, Stephen
Grant, William B.
Gray, Steven G.
Grignani, G.
Grivennikov, Sergei I.
Gronchi, Alessandro
Gu, Jian-Wei
Guenot, Dominique
Gupta, Kshitij
Gupta, Vineet
Gwenin, Christopher
Hall, Carolyn S
Harrison, Christine J.
Hartley, Rebecca S.
Hata, Yutaka
Hauser, T.
Haystead, Timothy A. J.
Hesson, Luke
Hiltermann, T. J. N.
Hoffman, Robert M.
Holdenrieder, Stefan
Hori, Tomohide
Howard, Steven P.
Hsieh, Ling-Ling
Hsieh, Yih-Shou
Huang, Tony Jun
Hubalek, Michael
Huh, Winston W.
Isayev, Olexandr
Jacobs, Andreas
Jacobs, Michael John
Jakob, Jens
Jessel, Nadia
Jiali, Han
Jin, Ge
Johansen, Pål
Judson, Ian
Juillerat-Jeanneret, Lucienne
Kalinina, Juliya
Karlic, Heidrun
Kashuba, Elena
Kavan, P.
Khan, Imran
Khazaie, Khashayarsha
Kim, Jung Ryul
Kirby, Brian
Klampfer, Lidija
Koga, Fumitaka
Komohara, Yoshihiro
Kondo, Akihiko
Kondo, Yutaka
Kong, Fan-Lin
Koren III, John
Krämer, Alwin
Krebs, Matthew G
Kumar, Arun
Kuo, Min-Liang
Kwok, Albert
Lahav, Meir
Lam, Yee Cheong
Lamy, Pierre-Jean
Lania, Luigi
Lantz, Olivier
Le, Lu Q.
Li, Min
Lianidou, Evi
Lieber, Michael R.
Lin, Cheng-Wen
Liška, Václav
Liu, Fei
Lo Muzio, Lorenzo
Loeb, David M.
Looyenga, Brendan D.
Lopez, Jose Luis
Lu, Xiongbin
Lugnier, Claire
Lukanova, Annekatrin
Lupold, S. E.
Ma, Lijun
Macciò, Antonio
Maciaczyk, Jaroslaw
Maher, Elizabeth
Makishima, Makoto
Marcucci, Fabrizio
Marples, Brian
Marschalek, Rolf
Masotti, Andrea
Masucci, Maria
Matsuno, Akira
Matta, Jamie L.
Mavria, Georgia
McLaughlin, Patricia J.
Mehta, Rajendra G.
Merav, Leiba
Messaritakis, Ippokratis
Mierke, Claudia Tanja
Molinari, Michele
Mustjoki, Satu
Nadiminty, Nagalakshmi
Nagaraj, Vinay J.
Nahon, Pierre
Nasser, Mohd
Nazarian, Javad
Nemunaitis, John
Niu, Gang
Nosho, Katsuhiko
O’Connell, Maria A.
O’Neill, Eric
Oceandy, Delvac
O’Connell, Maria A.
Okabe-Kado, Junko
Oliver, F. Javier
Olivier, Magali
Olsen, Christian
Oscier, D.
Overmeyer, Jean
Pajonk, Frank
Paller, Channing
Panchapakesan, Balaji
Pang, Jong-hwei S.
Pantel, Klaus
Pappas, Dimitri
Pego, Ana Paula
Pellat-Deceunynck, Catherine
Peng, Guang
Pennacchioli, Elisabetta
Pierceall, William E.
Pierga, Jean-Yves
Pradhan, Sriharsa
Price, David
Profirovic, J.
Prosperi, Ennio
Pullen, N. A.
Putnam, Chris H.
Quesenberry, P.
Ragno, Rino
Rak, Janusz
Ramnath, Nithya
Rassool, Feyruz
Ray, Amitabha
Redell, Michele
Reeves, Helen
Reigan, Philip
Reiko, Imai
Rich, Jeremy N.
Robbiani, Davide F.
Rochel, Natacha
Rosato, Antonio
Russell, Jeffrey
Ryan, Anderson
Santamaría, David
Sanz-Moreno, Victoria
Savaşan, Süreyya
Schuetze, Scott M.
Schuller, Hildegard M.
Schultz, Christopher J.
Schwartz, Gary
Scott, Jacob G.
Searle, Peter F.
Sheikh, Nadeem
Shui, Irene M.
Singh, Rajan
Slominski, Andrzej
Smith, Steven
Soto, José-Luis
Sowers, Lawrence C.
Stebbing, Justin
Stephenson, Sally-Anne
Stern, Stephan T.
Stoecklein, Nikolas
Strefford, J.
Studzinski, George P.
Su, Gang
Su, Wu-Chou
Suh, Nanjoo
Tabatabai, Ghazaleh
Tanaka, Fumihiro
Tapon, Nicolas
Terstappen, Leon W. W. M.
Thomas, Davidm
Thway, Khin
Tilki, Derya
Tilkorn, Daniel-Johannes
Tinhofer-Keilholz, Ingeborg
Tochner, Zelig
Torigoe, Toshihiko
Trabert, Britton
Trump, Donald L.
Tsao, Hensin
Tseng, Ching-Ping
Van Hall, Thorblad
Vik-Mo, Einar Osland
Virolle, Thierry
Vlahogianni, Thomais
Vona-Davis, Linda
Walker, Graeme
Walker, Paul
Wang, Guiren
Wang, Lidai
Wang, Marilene B.
Watts, Colin
Weljie, Aalim
Wheeler, Chris
White, John
Wu, Xiangwei
Xia, Hongping
Yamaguchi, Umio
Yamamoto, Katsuya
Yamamoto, Keiko Yamamoto
Yen, Katharine E.
Yodoi, Junji
Yoshino, T.
Young, Jason C.
Yu, Jian
Zauli, Giorgio
Zhang, Hongtao
Zhang, Xiao-Bing
Zhuang, Shougang
Ziman, Mel
Zwartkruis, Fried J.

